# Genetic data sharing and artificial intelligence in the era of personalized medicine based on a cross‐sectional analysis of the Saudi human genome program

**DOI:** 10.1038/s41598-022-05296-7

**Published:** 2022-01-26

**Authors:** Abdulmajeed F. Alrefaei, Yousef M. Hawsawi, Deyab Almaleki, Tarik Alafif, Faisal A. Alzahrani, Muhammed A. Bakhrebah

**Affiliations:** 1 Department of Biology, Genetic and Molecular Biology Central Lab, Jamoum University College, Umm Al-Qura University, Makkah, 21955 Saudi Arabia; 2 Research Centre, King Faisal Specialist Hospital and Research Centre, P.O. Box 40047, Jeddah, 21499 Saudi Arabia; 3 MBC: J04/ College of Medicine, Al-Faisal University, P.O. Box 50927, Riyadh, 11533 Kingdom of Saudi Arabia; 4 Department of Evaluation, Measurement, and Research, Umm Al-Qura University, Makkah, 21955 Saudi Arabia; 5 Computer Science Department, Jamoum University College, Umm Al-Qura University, Jamoum, 25375 Saudi Arabia; 6 Department of Biochemistry, Faculty of Science, Embryonic Stem Cells Unit, King Fahd Medical Research Center, King Abdulaziz University, Jeddah, 21589 Saudi Arabia; 7 King Abdulaziz City for Science and Technology (KACST), Life Science and Environment Research Institute, P.O. Box 6086, Riyadh, 11442 Saudi Arabia

**Keywords:** Computational biology and bioinformatics, Genetics, Medical research

## Abstract

The success of the Saudi Human Genome Program (SHGP), one of the top ten genomic programs worldwide, is highly dependent on the Saudi population embracing the concept of participating in genetic testing. However, genetic data sharing and artificial intelligence (AI) in genomics are critical public issues in medical care and scientific research. The present study was aimed to examine the awareness, knowledge, and attitude of the Saudi society towards the SHGP, the sharing and privacy of genetic data resulting from the SHGP, and the role of AI in genetic data analysis and regulations. Results of a questionnaire survey with 804 respondents revealed moderate awareness and attitude towards the SHGP and minimal knowledge regarding its benefits and applications. Respondents demonstrated a low level of knowledge regarding the privacy of genetic data. A generally positive attitude was found towards the outcomes of the SHGP and genetic data sharing for medical and scientific research. The highest level of knowledge was detected regarding AI use in genetic data analysis and privacy regulation. We recommend that the SHGP’s regulators launch awareness campaigns and educational programs to increase and improve public awareness and knowledge regarding the SHGP’s benefits and applications. Furthermore, we propose a strategy for genetic data sharing which will facilitate genetic data sharing between institutions and advance Personalized Medicine in genetic diseases’ diagnosis and treatment.

## Introduction

The completion of the human genome project in 2003 ushered in a revolution in genomic medicine^1,2^. Although the project initially cost over USD 3 billion, the value outcome was and continues to be much greater^3^. The massive data reward from the project is one of the major scientific breakthroughs of the twenty-first century, leading to remarkable advancements in genetics and the development of personalized medicine. With the growth in genomic technology and understanding of the value of genetics, multiple countries initiated genome projects such as the Encyclopedia of DNA Elements (ENCODE)^4^, the 1,000 Genomes Project (1KGP)^5^, UK 100,000 Genomes Project in 2012^6,7^, the UK Biobank Exome Sequencing Consortium (UKB-ESC)^8^, and the Saudi Human Genome Project (SHGP)^9^. Due to the high rate of consanguinity in Saudi Arabia (SA), which exceeds 60% of total marriages^10^, and large family sizes, SA is an ideal country in which to discover novel variants. Therefore, autosomal recessive diseases have become relatively common in SA, and several rare diseases have been recognized^10–12^. For example, congenital malformations, congenital heart disease (CHD), cystic fibrosis, Duchenne muscular dystrophy (DMD), hereditary recessive deafness, hereditary blindness and visual impairment, thalassemia and sickle cell disease have been reported frequently in SA^13^. Accordingly, many variants have been reportedly associated with genetic diseases in Saudi Arabian populations^13–15^.

In 2013, the SHGP was launched in SA by King Abdulaziz City for Science and Technology (KACST). The program was launched to herald a new era of personalized medicine and diagnosis of hereditary diseases^9^. The goals of the SHGP are to build a genetic database for Saudi citizens, prevent and limit genetic diseases, enable researchers and scientists, to benefit from the genetic information in the program, and determine genetic variants and use them to develop diagnostic prevention tools, to reduce the incidence of genetic diseases and improve community well-being. In addition, the initiative intends to create an advanced infrastructure in the fields of genomics and bioinformatics, with the goal of enhancing treatment approaches based on the genetic makeup of patients (https://shgp.kacst.edu.sa/index.en.html#program-objectives). With secure funding, devoted resources, and national determination, the SHGP created a target of sequencing 100,000 human genomes via seven satellite laboratories^9^. The cutting edge technology known as next-generation sequencing (NGS) has been used for clinically efficient and cost-effective readings of Saudi Human Genomes^16^. One outcome of the program is that more than 65,000 samples have been sequenced and 7,500 variants have been identified to date. Approximately, 3,000 of these variants are novel causative mutations directly associated with over 1,230 rare genetic disorders^17^. These results will allow for the prediction of the potential occurrence and reoccurrence of some rare genetic syndromes, thereby advancing curative approaches by proposing suitable medication determined by the patient’s genetic makeup^8,17,18^.

A national information base at the KACST was also established to store data on population variations and make this data available to clinicians in SA to enable future diagnostic and screening efforts^9,17^. The SHGP contains a specific genome database targeting the Saudi population. The database is collected from several servers and a large computer hosted by KACST. Eight satellite laboratories scattered across the kingdom are connected with a central server. Therefore, huge data are processed daily that are used by medical and research institutions to study genetic disorders and enhance health care. With the advancement of genomic technology, the massive genome data generated requires artificial intelligence (AI) algorithms for disease identification, diagnostic predictions, and suggested treatments [High-performance Medicine: The Convergence of Human and Artificial Intelligence]. With this massive data, clinicians may manually perform these tasks, but they require a large amount of time and efforts. While clinicians require much time to perform the tasks, AI based algorithms can perform these tasks within seconds.

AI is a sub-area of computer science which investigates and applies computational algorithms by mimicking humans to solve complex problems^19^. Currently, AI algorithms are applied to many tasks in health care, including human genomes^20^. Using supervised, semi-supervised, unsupervised learning, and evolutional manners, these algorithms can identify human genetic patterns and disorders from genetic data^20^. Specific AI algorithms known as deep learning can solve clinical diagnostic tasks, predict RNA-Seq profiles, and improve precision medicine for complex diseases using complicated and large-scale genomic databases^21^ .AI-based deep learning algorithms can also be applied for variant calling, variant classification and phenotype-to-genotype and genotype-to-phenotype mappings and classifications^19,20^. Deep learning algorithms are also currently being used for nucleotide sequence data in genomic applications^20^. Since AI algorithms use data, AI and data privacy are becoming tightly associated. However, employing AI-based algorithms without engaging policies and regulations can potentially endanger human genetic data privacy.

Data privacy is an important branch of data security. It mainly focuses on how the data are handled using policies including consents, notices, and regulatory obligations. Recently, genomics data has been collected, grown and stored for medical and research purposes to explore genome features and solve genetic problems manually and computationally by clinical practitioners and bioinformaticians, respectively^22,23^. Genomics data contains sensitive personal details about patients and their relatives^24–27^. Hence, the complexity of genetic data sharing and AI arises when researchers and physicians call for flexible data sharing policy. At the same time, public and patients are concerned about data confidentiality, privacy, discrimination, unethical misuse, and security.

Since the success of SHGP is highly dependent on awareness in the population, we studied the awareness, knowledge and attitude of the Saudi population toward the SHGP. The objectives of this study were to (1) assess public knowledge regarding genetic diseases, (2) investigate the level of public awareness toward the SHGP, (3) study the public awareness of genetic data privacy resulting from the SHGP and (4) analyse the public attitude and knowledge regarding the role of AI in the management of privacy and analysis of SHGP genetic data. The outcome of this study will help decision-makers involved in the SHGP in strategic planning for public communication and the implantation of the SHGP findings.

## Methodology

### Study design

This cross-sectional study was conducted using an online questionnaire to explore the Saudi public’s awareness, knowledge, and attitude toward the SHGP and genetic data privacy and AI use in the SHGP data among the Saudi population.

### Ethical approval

The questionnaire was distributed after obtaining ethical approval from the Institutional Review Board (IRB) of King Abdulaziz City for Science and Technology (KACST) (IRB; 200,100). The IRB committee approved all protocols, and the relevant regulations and guidelines were followed.

### Subject recruitments

An electronic format of the questionnaire consisted of an introduction of the study’s aims, including the importance of voluntary contribution in the study and a consent statement. The questionnaire was distributed via different social media platforms in Saudi Arabia including Twitter, WhatsApp and Telegram. Saudis are very active in these platforms, for example, they ranked seventh in the world in terms of Twitter users (12.7 million). All Saudi citizens aged ≥ above or equal than 18 years were targeted to participate in the study. More than 844 responses were received, and exclusion criteria were (a) None-Saudi, (b) less than 18 years old, and (c) incomplete responses.

### Study instruments

The questionnaire was designed, validated and the electronic format was created using Google Forms. The validated version of the survey consisted of six sections: (1) social and demographic information including, age, gender, educational level, and nationality, (2) participants’ awareness of genetic diseases (6 items), (3) participants’ awareness of the SHGP (8 Items), (4) Saudi citizens’ knowledge and attitude of genetic data privacy of the SHGP (9 Items), (5) attitude toward the use of AI in the genome and the privacy management of genetic data (6 Items) and (6) attitude toward sharing genetic data in scientific research (2 Items).

### Statistical analysis

All responses were imported and categorized into Excel spreadsheets for descriptive and statistical analyses. The statistical software programs SAS (version 9.4) and SPSS (version 25) were used to perform t-tests and multivariate statistics ANOVA to analyse several significant variables, including the level of public knowledge and awareness regarding the SHGP, genetic data privacy/sharing and AI use. Statistical significance was considered at a P value of less than 0.05 for all analyses.

### Excluding responses

We excluded 40 respondents who chose “non-Saudi” since we could not confirm if they lived in the SA.

### Informed consent statement

Informed consent was obtained from all subjects involved in the study.

## Results

### Participant characteristics

A total of 844 participants completed the survey, of which 40 non-Saudi were excluded from the analysis. Therefore, we included 804 responses in the present analysis. All analysed responses were Saudi citizens, with the gender distribution being 38.4% female (309/804) and 61.6% male (495/804). Almost half (42%, 345/804) of the participants were 18 to 28 years of age, and 26.1% were 28 to 38 years of age (211/804). More than half of the participants, 55.1%, held a bachelors’ degree (443/804). Almost half of the participants were single (47.8%), and a slightly higher percentage were married (49.6%). A detailed demographic data of the survey respondents as shown in Table 1 and Supplementary Fig. 1.

**Table 1:** Demographic data of study participants (*n* = 804).

	*n*	%
**Age**		
18 to less than 28	345	42.9
28 to less than 38	211	26.2
38 to less than 48	145	18.0
48 to less than 58	66	8.20
greater than 58	37	4.60
**Gender**		
Male	495	61.6
Female	309	38.4
**Education level**		
Secondary school	86	10.7
Bachelor	443	55.1
Postgraduate	232	28.9
Other	43	5.30
**Marital status**		
Single	384	47.8
Married	399	49.6
Divorced	17	2.10
Widowed	4	0.50
**Nationality**		
Saudi	804	100

### Awareness of genetic diseases among participants

The SHGP was launched to study the causes of the high prevalence of genetic disorders and detect rare inherited diseases among Saudi citizens. Therefore, we investigated the level of public awareness about different aspects of genetic diseases in SA as shown in Table 2 and Supplementary Fig. 2. Approximately 74.3% of study participants were aware of the high prevalence of genetic diseases among Saudis. Almost all participants (93.8%) knew genetic diseases negatively impact affected individuals and their families. Most participants (90.2%) were aware of the role of consanguinity in the increase of genetic disease incidence. Interestingly, only 19.8% of participants had undergone genetic testing, but nearly all participants (95.6%) had a positive attitude and high awareness of the importance of pre-marital screening in reducing the prevalence of inherited diseases. Further analysis revealed that overall awareness of genetic diseases was significantly higher in females than males (*p* = 0.0094) as shown in Table 3.

**Table 2:** Study participants awareness of genetic diseases among study participants.

Questionnaire item	Yes		No	
	*n*	%	*n*	%
1. The prevalence of genetic diseases is high among members of Saudi society	597	74.3	207	25.7
2. Genetic diseases have a negative impact on the patient and his/her family	754	93.8	50	6.20
3. Consanguinity is one of the causes for increasing rates of genetic diseases	725	90.2	79	9.80
4. I have previously done a test for genetic diseases	159	19.8	645	80.2
5. Pre-marital screening will reduce the prevalence of genetic diseases	769	95.6	35	4.40

**Table 3:** Comparison (t-test) of variables by gender across the components of the questionnaire.

Questionnaire categories			Mean	df	t	*p*-value
1. Awareness of genetic diseases	Gender	Male	5.044	802	1.12	0.0094
		Female	5.420			
2. Awareness and attitude toward the SHGP	Gender	Male	4.504	802	1.43	< 0.0001
		Female	5.181			
3. Society knowledge and attitude and data privacy of the SHGP	Gender	Male	29.989	802	1.21	0.2265
		Female	30.571			
4. Attitude toward the use of AI in the privacy of genetic data and the SHGP	Gender	Male	4.825	802	0.54	0.5911
		Female	4.871			

*: statistically significant *p*-value < 0.05.

### Awareness and attitude toward the SHGP

Despite the massive media campaign launched in 2021 about the SHGP, only 40.5% of study respondents had heard of the SHGP as shown in Table 4. Moreover, 73.8% of participants were not aware of the benefits and applications of the SHGP. The vast majority of participants (82.1%) assumed that the SHGP would document the first genetic map of Saudi citizens. Approximately 86.3% of respondents chose “yes” for the possible contribution of the SHGP to gene therapy development. Furthermore, 87.2% of participants had a positive attitude toward the contribution of the SHGP in the localization of genomic techniques and genetic research. Only 4.6% of participants were among the sample donors in the program, but 68.8% of them were willing to participate. More than 80% of participants were optimistic about the contribution of the SHGP in decreasing the prevalence of genetic diseases in Saudi society. Further analysis showed that awareness and attitude toward the SHGP were not statistically significant compared to the effect of status and age. However, educational attainment was significantly correlated to program awareness (F (3, 801) = 6.49, *p* = 0.0002). People with a postgraduate degree (*p* = 0.0005, M = 5.164) were more aware of the SHGP than people with a bachelor’s or another degree (M = 4.218).

**Table 4:** Study participants awareness and attitudes toward the SHGP.

Questionnaire item	Yes		No	
	*n*	%	*n*	%
1. Have you heard about the Saudi Human Genome Program?	326	40.5	478	59.5
2. Have you heard about the benefits and applications of the Saudi Human Genome Program?	211	26.2	593	73.8
3. The Saudi Genome Program documents the first genetic map of Saudi society	660	82.1	144	17.9
4. The Saudi Genome Program contributes to developing gene therapy applications	694	86.3	110	13.7
5. The Saudi Genome Program contributes to the localization of genomic techniques and genetic research	701	87.2	103	12.8
6. I was among the sample members in the Saudi Human Genome Project	37	4.60	767	95.4
7. I am ready to participate in the Saudi Human Genome Program	552	68.7	252	31.3
8. The Saudi Human Genome Project will contribute to inventorying genetic diseases in Saudi society	646	80.3	158	19.7

### Knowledge and attitude toward genetic data privacy of the SHGP

Nine items in the survey questionnaire focused on examining the level of knowledge and attitudes toward genetic data privacy of the SHGP (Table 5, Supplementary Table 1). Approximately 38.8% of responses showed the lowest level of knowledge toward the process of preserving and managing genetic data in the SA, and 25.1% of them had a medium level of knowledge. Approximately 43.8% of participants did not know the institutions responsible for storing Saudi genetic data, while 20.4% showed a medium level of knowledge. Regarding the management of genetic data with high privacy, 28.1% of participants expressed a medium level of knowledge, while 33.3% chose the highest level. Furthermore, nearly the same responses were detected regarding the high security of genetic data of the SHGP: 28.1% chose a medium level and 33.3% the highest level. Additionally, 56.6% of responses exhibited the lowest level of attitude toward sharing the genetic data without privacy protection, and only 15.8% of them chose the medium level.

**Table 5:** Public knowledge and attitudes toward data privacy and security of the SHGP.

Questionnaire item	Lowest level				Highest level
	1	2	3	4	5
*P*	%	%	%	%	%
1. I know the process for preserving and management of genetic data in Saudi Arabia	38.8	15	25.1	7.7	13.3
2. I know the bodies responsible for preserving Saudi genetic data	43.8	18.5	20.4	6.6	10.7
3. The genetic data of Saudi society is managed with high privacy	12.9	9.5	26.2	17.9	33.6
4. The genetic data resulting from the SHGP receive a high degree of security	14.3	7.7	28.1	16.5	33.3
5. I support the dissemination of genetic data without the protection of privacy	56.5	12.9	15.8	5.3	9.5
6. Have you heard about the importance of privacy and the security of genetic data?	28.4	12.7	20.6	12.3	26.2
7. I support the need to obtain the consent of the patient before sharing his/her genetic data	4.7	1.9	7.5	6.3	79.7
8. I support the need for a general policy for the privacy of genetic data	4.1	2.1	8.7	7	78.1
9. It is important to hold seminars to introduce the importance of privacy and security of genetic data	3.4	1.5	9	10.8	75.4

**Figure 1:**
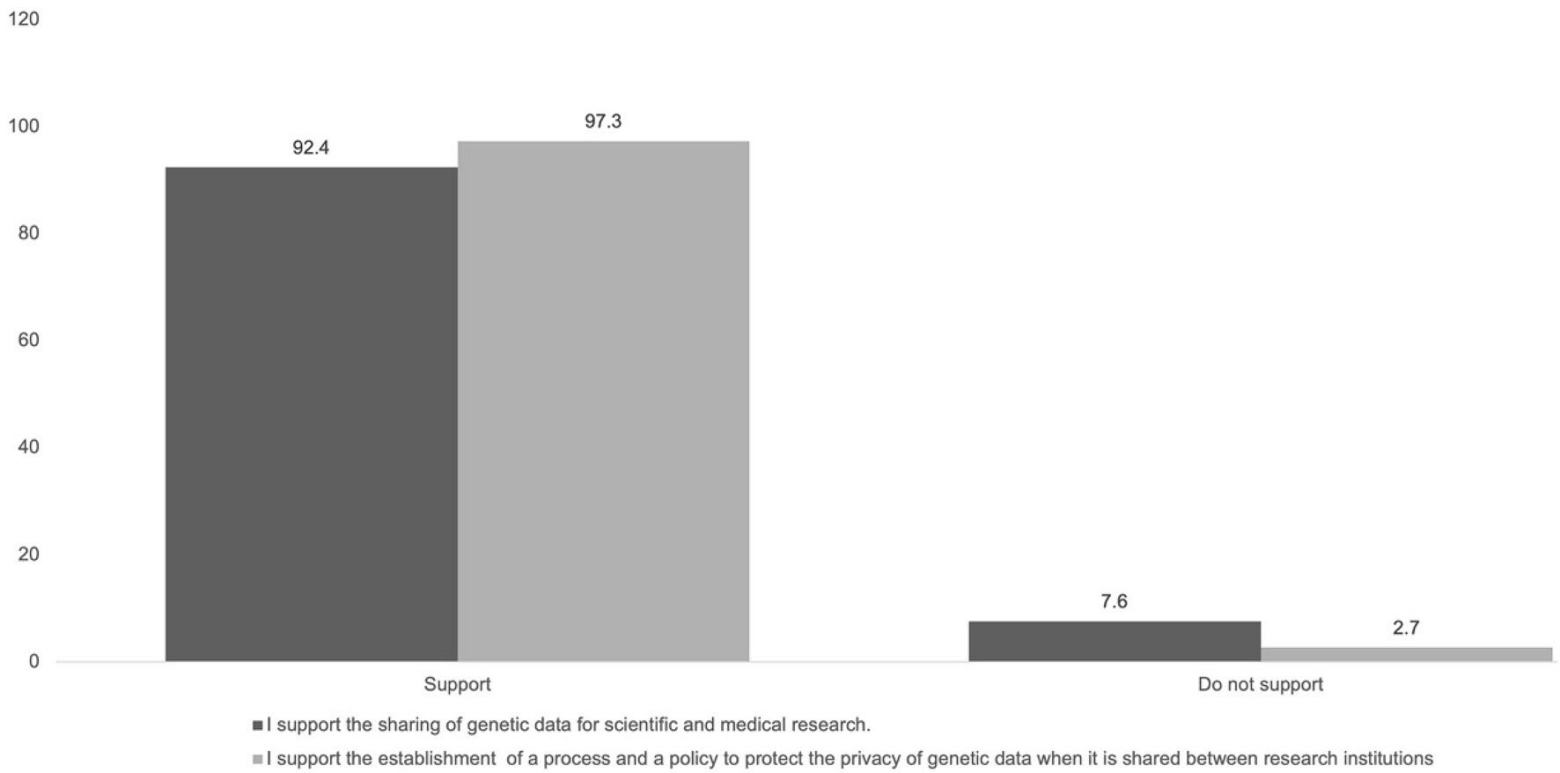
Study participant responses regarding genetic data sharing.

Interestingly, there was uncertainty regarding the level of knowledge of the importance of the privacy and the security of genetic data, as the responses were divided between the lowest level (28.4%), medium level (20.6%), and highest level (26.2%); the remainder was not sure. A majority of participants (79.7%) felt the highest level of positive attitude and support for obtaining the patient’s consent before sharing their genetic data. Similarly, the highest level of attitude and support were reported regarding the need for a general policy for the privacy of genetic data (78.1%). Importantly, most participants (75.4%) showed the highest level of positive attitude toward the importance of organizing seminars to introduce the knowledge related to privacy and security of genetic data. Positively, most participants supported genetic data sharing in scientific and medical research and establishing a national policy to protect genetic data privacy when shared between Saudi institutions (Fig. 1).

### Attitudes toward the use of AI in the privacy of genetic data

As massive genetic data are generated and become big data, rapid and accurate analysis is required AI to provide clinical reports for health diagnoses or other related tasks in research or medical fields. Thus, we investigated the attitude and opinions of Saudi society about the involvement of AI in the privacy of genetic data and its role in SHGP data analysis (Table 6). Surprisingly, 92.8% of participants agreed that AI could be used to analyse genetic data. Furthermore, most participants (80.6%) agreed to AI contributing to solving genetic disorders. A vast majority of participants (90.7%) agreed that AI technologies could provide solutions to ensure the privacy of genetic data. Most participants (88.8%) agreed with employing AI in managing the privacy of genetic data. However, the participants were divided regarding the threat of AI use in the privacy of genetic data as 41.2% chose “agree” and 58.8% chose “do not agree”. Positively, 90.7% of participants agreed that AI could be used in the SHGP.

**Table 6:** Study participant attitudes toward the use of AI in the privacy of genetic data in the SHGP.

Questionnaire item	Agree		Do not agree	
	*n*	%	*n*	%
1. Artificial intelligence can be used to analyse genetic data	746	92.8	58	7.2
2. Artificial intelligence contributes to solving genetic disorders	648	80.6	156	19.4
3. Artificial intelligence technologies can provide solutions that ensure the privacy of genetic data	729	90.7	75	9.3
4. Artificial intelligence can be employed in managing the privacy of genetic data	714	88.8	90	11.2
5. The use of artificial intelligence is a threat to the privacy of genetic data	331	41.2	473	58.8
6. Artificial intelligence can be used in the SHGP	729	90.7	75	9.3

The statistical analysis showed that the attitude toward using AI in the SHGP was significantly different by educational level (F (3, 801) = 4.68, *p* = 0.0030). Participants with a postgraduate degree (*p* = 0.0110, M = 5.043) had more attitude toward using AI in the privacy of genetic data and the SHGP than those with a bachelor’s degree (M = 4.574). Moreover, there was a statistically significant difference by marital status (F (3, 801) = 7.28, *P* < 0.0001). People who were married (*P* < 0.0001, M = 5.040) had more attitude toward the use of AI in the privacy of genetic data and the SHGP than single people (M = 4.574). Furthermore, specific age groups were significantly different (F (4, 800) = 4.35, *P* = 0.0018). People who were 38 years old to less than 48 years old (*P* = 0.0100, M = 5.055) had more attitude toward the use of AI in the privacy of genetic data and the SHGP than people who were of age 18 years old to less than 28 years old (M = 4.666).

## Discussion

The SHGP was recently established to detect and study the causes of genetic disorders. In this study, we found that most participants were aware of the high prevalence of genetic diseases among Saudis (Table 2). Most participants considered consanguinity as a factor in genetic diseases. Nearly, all participants had a positive attitude and sufficient awareness of pre-marital screening in reducing the prevalence of inherited diseases. These results are consistent with our previous study such that other reports showed the Saudi community has a high level of awareness toward genetic testing^28–31^. One possible reason for these positive findings, is that in 2002, the Saudi government passed a law requiring pre-marital genetic testing^32^. Interestingly, the results of our study also revealed that females had significantly higher awareness of genetic diseases than males.

We then examined the awareness and attitude toward the SHG, and found inadequate awareness about the SHGP and its benefits and applications (Table 4). Thus, there is a need for greater efforts to educate people about the SHGP and human genome in general. Furthermore, we documented that a high parentage of participants assumed that the SHGP would establish the first genetic map of the Saudi community. There is a positive attitude among the responses regarding the contribution of the SHGP in gene therapy and the localization of genomic techniques. Moreover, the responses showed encouraging results (68.8%) in willingness to participate in the SHGP sample collection initiative. The participants were generally optimistic about the SHGP outcomes, potentially lowering the prevalence of genetic diseases and their negative impacts. In addition, the analysis revealed that knowledge and attitudes concerning the SHGP were not statistically significant in comparison with the effect of status and age. However, there was a significant correlation between educational attainment and awareness level as people with postgraduate degrees were more aware of the SHGP than those with bachelor's degrees.

Regarding the level of knowledge and attitude toward genetic data privacy and management of the SHGP data, an insufficient level of knowledge was reported (Table 5). The participants did not have enough knowledge regarding the process of preserving and managing genetic data, and less than half did not know the institutions responsible for storing the genetic data in the SA. Regrading genetic data privacy and security, uncertainty and a low level of knowledge were detected among respondents. A high rate of concern about patient privacy was reported as most participants called for informed consent before sharing their genetic data. Similarly, the highest level of attitude and support was detected for applying general policy to genetic data privacy. Importantly, most responses exhibited the highest level of positive attitude toward the importance of organizing seminars to introduce the knowledge related to privacy and security of genetic data. We noticed some contradictions responses in a few questions related to genetic data privacy and genetic data sharing. For instance, 43.8% of participants did not know the institutions responsible for storing Saudi genetic date while 33.6% of them chose the highest level of knowledge regarding the management of genetic data with high privacy in the SA (Table 5). These contradictions could be a result of low level of knowledge and awareness about these issues among the participants.

**Figure 2:**
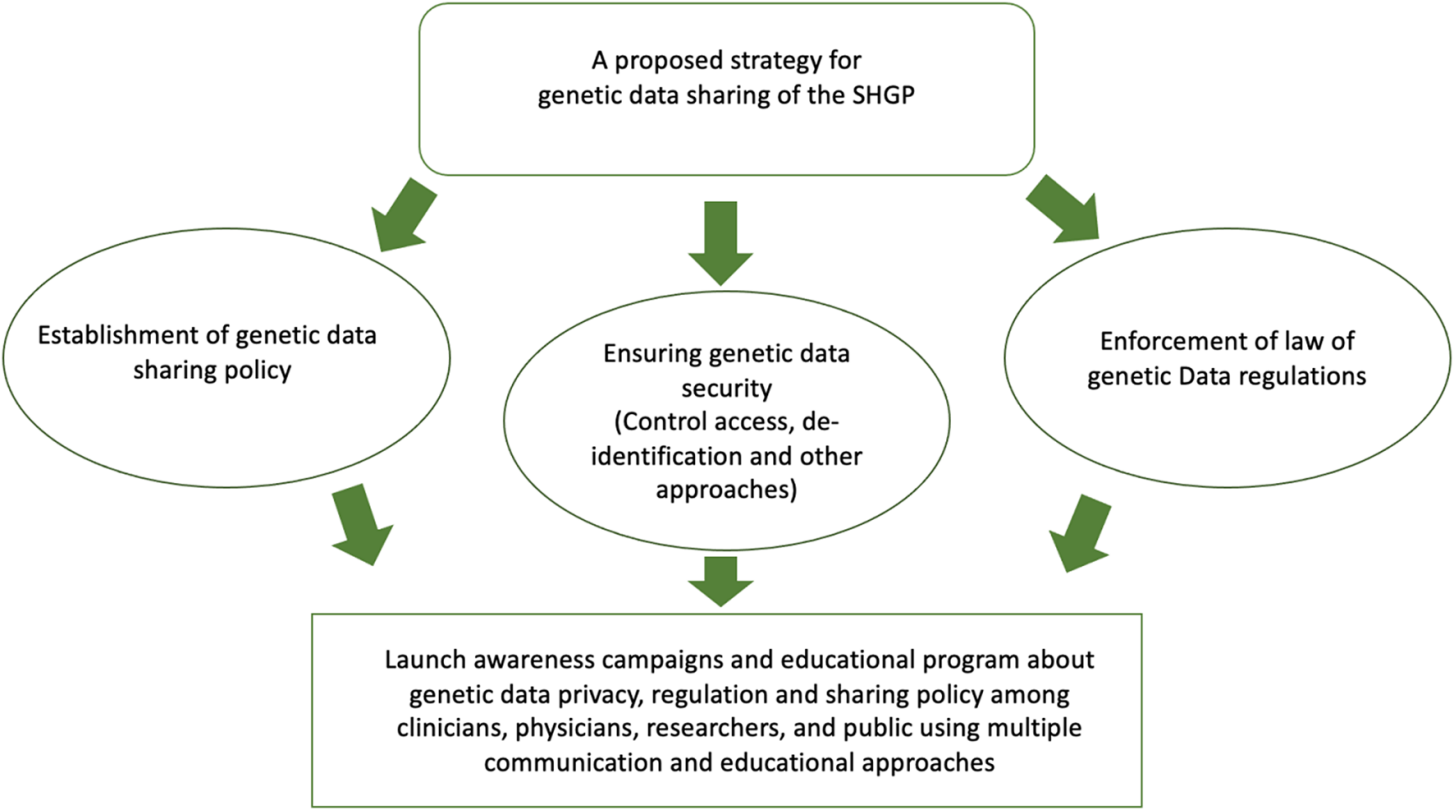
Proposed strategy for genetic data sharing of the SHGP. First, a national policy for genetic data sharing should be established. Second, advanced technologies should be used to ensure genetic data security and privacy. Third, laws governing genetic data regulation must be enforced. Finally, national awareness campaigns and educational programs should be launched among clinician, physicians, researchers, and the general public.

Positively, most participants supported genetic data sharing in scientific and medical research and the establishment of a national policy to protect the privacy of genetic data when it is shared between Saudi institutions (Fig. 1). We found that the public support genetic data sharing if their privacy and personal information are secured. Consistent with this, a study conducted in Riyadh, the SA, showed that 78.4% of the participants are in favour of building a database of hereditary diseases and managed by the government^28^. However, several reports have shown that the public is always concerned about data misuse, and being identified, and stigmatized with genetic diseases^23,24,26,27^. For example, surveys were conducted in Pennsylvania (the United States) and Bavaria (Germany) about Personalized Medicine showed that most participants were worried about genetic data misuse^33^. Notwithstanding, the general public trusts researchers in the hope of finding cures for complex diseases. Based on these findings, we propose a strategy for sharing the SHGP data that ensure the privacy and security of genetic data (Fig. 2). The sharing of genetic data will broaden opportunities for researchers and medical practitioners to accelerate gene therapy discovery, improve the diagnosis of genetic diseases and develop personalized medicine for patients^27,34,35^. Consistent with this idea, other investigators have called to establish a national genomic data-sharing policy in the SA that allows data to be freely shared among institutions to enhance bio-marker discovery and computational biology analysis, improving the treatment of genetic disease complications^34,35^. A lack of a genetic data sharing policy will limit the use, access, and analysis of the SHGP data. A genetic data sharing policy will regulate the privacy of genetic data if it is shared with a third party and how it is shared. Also, the policies should regulate how the genetic data is collected, stored, and provided in its legal state.

We also further investigated public attitudes toward the use of Al in the analysis of genetic data and privacy regulation in the SHGP (Table 6). We reported the highest positive attitude toward AI use in genetic data analysis. Furthermore, most participants trusted the ability of AI to solve genetic disorders. In terms of the privacy of genetic data, a vast majority of responses indicated that AI technologies could ensure and manage privacy. However, the participants had divided opinions regarding the threat of AI use in privacy regulations. Almost all participants had positive attitudes toward the use of AI in the SHGP. Furthermore, our statistical analysis revealed that the attitude toward using AI in the SHGP was significantly different by educational level (F (3, 801) = 4.68, *p* = 0.0030). Participants with a postgraduate degree (*p* = 0.0110, M = 5.043) had a higher positive attitude on employing AI in the privacy of genetic data and the SHGP than those with a bachelor’s degree (M = 4.574). Moreover, People aged 38–48 (*P* = 0.0100, M = 5.055) had a more positive attitude about the use of AI in the privacy of genetic data.

Surprisingly, participants showed a higher level of positive attitude and knowledge toward Al applications than the SHGP and its benefits. This result could be because Al is trending now in the SA. More specifically, the government has established Saudi Data and Artificial Intelligence Authority (SDAIA). In addition to this, several media campaigns have presented information about AI and its Applications^36^.

Despite some concerns about AI use in health care and genomic data, such as inaccuracies, discrimination, and bias in the database, AI algorithms will revolutionize genomics and proteomics data analysis, improving precision medicine in genetic disease diagnosis and treatment^23,37^. AI algorithms, more specifically, deep learning based algorithms are currently being employed in clinical diagnosis and analysis of complex and large-scale genomic databases. However, AI based algorithms may require huge databases to train to improve genomic data analysis and drug discovery. Therefore, genetic data sharing will definitely improve the use of AI in the SHGP and Personalized Medicine. Furthermore, AI and privacy technologies could provide solutions for genetic data sharing, for example, cryptography, differential privacy and other approaches^23,24,38,39^.

In the current study, we analysed and assessed Saudi public awareness, knowledge, and attitudes toward the SHGP, genetic data privacy and the role of AI in the management of privacy and the analysis of genetic data. To the best of our knowledge, this study is the first population-based survey of Saudi public awareness and knowledge toward the SHGP. We anticipate that the outcome of this study can help decision-makers involved in SHGP management and genetic data regulation plan public communication strategically, implement SHGP findings, and establish a national genetic data sharing policy.

## Conclusion

This study provides insights regarding the Saudi society’s awareness, knowledge, and attitude towards the SHGP, the sharing and privacy of genetic data resulting from the SHGP, and the role of AI in managing privacy and analysing genetic data. We reported moderate awareness and attitude towards the SHGP and minimal knowledge regarding its benefits and applications. In addition, a low level of knowledge was observed regarding sharing and privacy of genetic data. A generally positive attitude was found towards the outcomes of the SHGP and genetic data sharing for medical and scientific research. Furthermore, the highest level of knowledge was detected regarding AI use in genetic analysis and privacy regulations. We identified gender, status and educational level as important factors in public awareness and knowledge of the SHGP. Furthermore, we proposed a strategy for genetic data sharing in Saudi Arabia. We recommend that awareness campaigns and educational programs be launched by institutions that manage the SHGP to increase and improve public awareness and fill the knowledge gaps regarding these issues.

## Supplementary Information

Supplementary Information.

## Data Availability

Correspondence and requests for materials should be addressed A F Alrefaei.
