# Supplementary material for: Genetic data sharing and artificial intelligence in the era of personalized medicine based on a cross‐sectional analysis of the Saudi human genome program
Source: Sci Rep. 2022 Jan 26;12:1405. doi: 10.1038/s41598-022-05296-7 (PMC8791994; doi:10.1038/s41598-022-05296-7)
Supplement: Supplementary file 1 — Supplementary Information. [file 41598_2022_5296_MOESM1_ESM.docx]

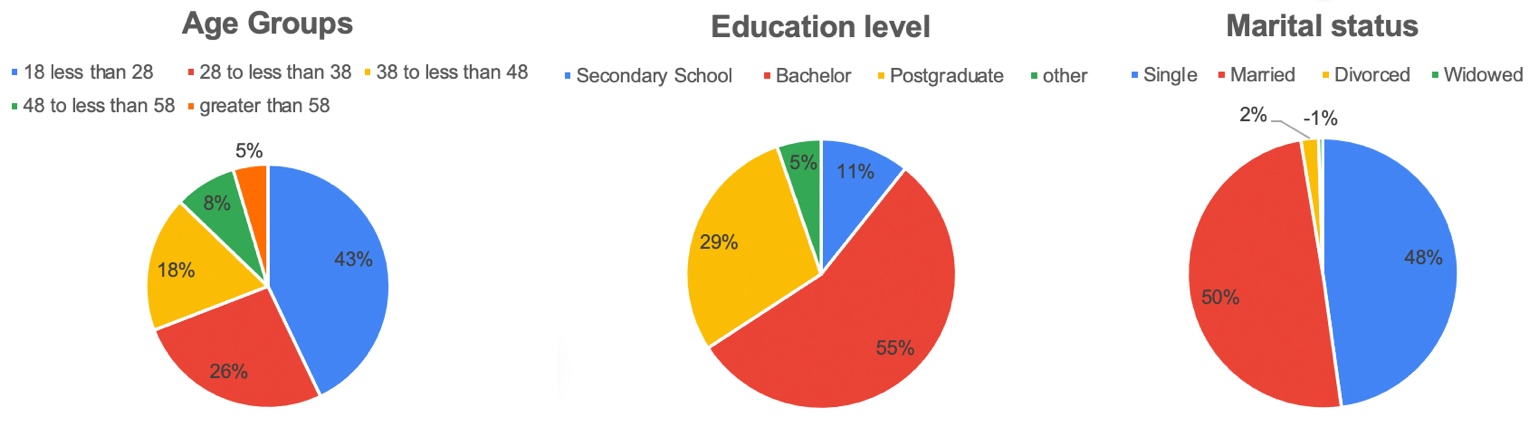


**Supplementary Figure 1 shows** demographic percentage of study participants (n = 804).

**Supplementary Figure 2** shows the participants’ responses regarding common diseases in their families.

**Supplementary Table 1** shows participants’ responses in number (N) regarding the knowledge and attitude of data privacy of the Saudi Genome Program.

| **4-Society and data privacy of the Saudi Genome Project** | **Lowest level** |  | | | **Highest level** |
| --- | --- | --- | --- | --- | --- |
|  | **1** | **2** | **3** | **4** | **5** |
|  | N | N | N | N | N |
| I Know the process for preserving and management of genetic data in Saudi Arabia | 312 | 121 | 202 | 62 | 107 |
| I Know the **bodies** responsible for preserving Saudi genetic data | 352 | 149 | 164 | 53 | 86 |
| The genetic data of the Saudi society is managed with high privacy | 103 | 76 | 211 | 144 | 270 |
| The genetic data resulting from the Saudi Human Genome Project receive a high degree of security | 115 | 62 | 226 | 133 | 268 |
| I support the **dissemination** of genetic data without protection of privacy | 454 | 104 | 127 | 43 | 76 |
| Have you heard about the importance of the privacy of the security of genetic data | 228 | 102 | 166 | 99 | 209 |
| I support the need to obtain the consent of the patient before sharing his genetic data | 37 | 15 | 60 | 51 | 641 |
| I support the need for a general policy for the privacy of genetic data | 33 | 17 | 70 | 56 | 628 |
| It is important to hold seminars to introduce the importance of privacy and security of genetic data | 27 | 12 | 72 | 87 | 606 |
